# Different Wines from Different Yeasts? “*Saccharomyces cerevisiae* Intraspecies Differentiation by Metabolomic Signature and Sensory Patterns in Wine”

**DOI:** 10.3390/microorganisms9112327

**Published:** 2021-11-10

**Authors:** Fanny Bordet, Chloé Roullier-Gall, Jordi Ballester, Stefania Vichi, Beatriz Quintanilla-Casas, Régis D. Gougeon, Anne Julien-Ortiz, Philippe Schmitt Kopplin, Hervé Alexandre

**Affiliations:** 1 Université Bourgogne Franche-Comté, AgroSup Dijon, PAM UMR A 02.102, F-21000 Dijon, France-Institut Universitaire de la Vigne et du Vin (IUVV), Rue Claude Ladrey, BP 27877, CEDEX, 21078 Dijon, France; Chloe.Roullier-Gall@u-bourgogne.fr (C.R.-G.); regis.gougeon@u-bourgogne.fr (R.D.G.); rvalex@u-bourgogne.fr (H.A.); 2 Lallemand SAS, 19 Rue des Briquetiers, CEDEX, 31700 Blagnac, France; ajulien@lallemand.com; 3 Centre des Sciences du Goût et de l’Alimentation, AgroSup Dijon, CNRS, INRA, Université Bourgogne Franche-Comté, 21000 Dijon, France; jordi.ballester@u-bourgogne.fr; 4 Food Science and Gastronomy Department, University of Barcelona, Nutrition, INSA (Institut de Recerca en Nutricio I Seguretat Alimentaria), 08921 Santa Coloma de Gramenet, Spain; stefaniavichi@ub.edu (S.V.); beatrizquintanilla@ub.edu (B.Q.-C.); 5 DIVVA (Développement Innovation Vigne Vin Aliments) Platform/PAM UMR, IUVV, Rue Claude Ladrey, BP 27877, CEDEX, 21078 Dijon, France; 6 German Research Center for Environmental Health, Research Unit Analytical BioGeoChemistry, Helmholtz Zentrum München, D-85764 Neuherberg, Germany; schmitt-kopplin@helmholtz-muenchen.de

**Keywords:** yeast, *Saccharomyces cerevisiae*, Chardonnay wine, metabolomic, volatile compounds, sensory analysis

## Abstract

Alcoholic fermentation is known to be a key stage in the winemaking process that directly impacts the composition and quality of the final product. Twelve wines were obtained from fermentations of Chardonnay must made with twelve different commercial wine yeast strains of *Saccharomyces cerevisiae*. In our study, FT-ICR-MS, GC-MS, and sensory analysis were combined with multivariate analysis. Ultra-high-resolution mass spectrometry (uHRMS) was able to highlight hundreds of metabolites specific to each strain from the same species, although they are characterized by the same technological performances. Furthermore, the significant involvement of nitrogen metabolism in this differentiation was considered. The modulation of primary metabolism was also noted at the volatilome and sensory levels. Sensory analysis allowed us to classify wines into three groups based on descriptors associated with white wine. Thirty-five of the volatile compounds analyzed, including esters, medium-chain fatty acids, superior alcohols, and terpenes discriminate and give details about differences between wines. Therefore, phenotypic differences within the same species revealed metabolic differences that resulted in the diversity of the volatile fraction that participates in the palette of the sensory pattern. This original combination of metabolomics with the volatilome and sensory approaches provides an integrative vision of the characteristics of a given strain. Metabolomics shine the new light on intraspecific discrimination in the *Saccharomyces cerevisiae* species.

## 1. Introduction

Targeted approaches are mostly used for metabolite analysis in oenology. However, they do not enable the study of the diversity of metabolites in a matrix, so only a limited number of compounds is considered. These approaches contribute to answering a specific question, while untargeted approaches allow a holistic perspective. Non-targeted approaches using high-resolution mass spectrometry (uHRMS) provide a more comprehensive view of the metabolome. Thanks to these high-throughput analyses, it is possible to account for the chemical diversity of a complex matrix and bring to light not only known compounds but also previously unidentified compounds. Already frequently used in the fields of medicine, agro-environment, and nutrition, uHRMS has also been applied since the early 2000s to the wine matrix, with the development of the concept of “oenomics” [1], which covers different aspects. Vaclavik et al. [2] were able to discriminate three red wines according to their variety by wine-metabolome analysis. Wines can also be discriminated by their biogeographic origins [3,4]. Moreover, it was shown that this approach could be used to authenticate wines and evaluate their quality [5]. On the other hand, the impact of ageing [4,6] and the modification of the matrix by oxidation are topics that are widely addressed by this metabolomic approach [7,8,9,10].

Besides oenomics, metabolomics have been applied for over a decade to understand the metabolism of microorganisms [11,12]. Metabolomics can provide, at a given moment, a representation of the cell phenotype according to genetic background and its expression under specific environmental conditions, offering indications of possible biochemical regulations that cannot be quantified by other omics approaches [13]. High-resolution mass spectrometry (HRMS) is used to profile and globally describe the non-volatile metabolome changes associated with metabolic conditions based on the simultaneous measurement of several thousand signals due to high sensitivity, resolution, and mass accuracy [14].

The impact of microorganisms on different matrices, including wine, was addressed in studies by Schmitt-Kopplin et al. and Garcia et al. [15,16]. Thus, metabolomics has made it possible to discriminate between wines produced by different yeasts or yeast couples. Roullier-Gall et al. were able to distinguish several finished wines with a characteristic composition, strain, or mix of strains used for fermentation [17]. This approach also allowed us to highlight differences in metabolism between different species, as well as within the same species of yeast, under specific conditions. For example, different strategies developed by two *Saccharomyces* species for cold resistance were observed on the level of various metabolisms, such as lipid metabolism and the shikimate pathway [18]. Additionally, differences were reported within the *Saccharomyces cerevisiae* species, notably in the volatile compounds formed [19,20,21]. These differences probably reflect differences in the metabolism of the strains that have not been explored. The innovative aspect of our study is to investigate, for the first time, the metabolomic differences among different strains belonging to the same species. Indeed, we demonstrated that each of the twelve studied strains possess a specific non-volatile metabolomic signature that allows us to discriminate them and address intraspecific diversity. We novelly combined uHRMS, a powerful tool, with different targeted approaches to develop an integrative vision of this intraspecific diversity within the species. Furthermore, the monitoring of oenological parameters and of the growth of yeasts in fermentation were associated with a targeted approach to identify the volatilome expressed at the end of alcoholic fermentation. Additionally, a sensory analysis of each of the associated wines was performed.

## 2. Materials and Methods

### 2.1. Yeast Strains

Twelve commercial strains of *Saccharomyces cerevisiae* (Lallemand Inc, Montreal, QC, Canada) were selected for this study. Each strain was supplied as an ADY and stored at 4 °C once opened. These strains were coded S1 to S12.

### 2.2. Growth and Fermentation Conditions

Each strain was rehydrated from ADY stock and then diluted at 0.1% (*v*/*v*) concentration in 150 mL of YPD medium (0.5% (*w*/*v*) yeast extract, 1% (*w*/*v*) bactopeptone, 2% (*w*/*v*) glucose, and 0.02% (*w*/*v*) chloramphenicol) in 250 mL Erlenmeyer flasks. After incubation at 28° C with stirring (150 rpm) for 18 h, the second culture, in 150 mL of pasteurized Chardonnay must, filtered on a 0.22 µm membrane was performed in Erlenmeyer flasks (250 mL) at 28 °C in static mode for 18 h. These later cultures were used to inoculate pasteurized Chardonnay must containing 226.6 g/L glucose/fructose, pH 3.92, and 343.1 mg/L total assimilable nitrogen, at 1 × 10^6^ viable cells/mL. These fermentations were performed in 2 L bottles containing 1 L of inoculated must covered with sterile cotton wool. For each strain, assays were conducted in four biological replicates at 20 °C without stirring. The end of fermentation was considered as the total depletion of sugars.

### 2.3. Monitoring of Biomass Growth

Cell viability in preculture and during fermentation was determined by flow cytometry. The fluorochrome used was cFDA (5–6 carboxyfluorescein diacetate) (Invitrogen, Molecular Probes, ThermoFisher Scientific, Illkrich, France) dissolved in acetone at a concentration of 1500 µM [22]. We added 3 µL of cFDA to 100 µL of diluted yeast suspension in Mc Ilvain buffer (100 mM citric acid, 200 mM Na_2_HPO_4_, pH 4). Samples were incubated in the dark 20 min before measurement. Flow cytometry was performed with a BD Accuri C6 flow cytometer, and the resulting data were processed using the BD Accuri C6 software. For each sample, 20 µL was analyzed at 34 µL/min. The FSH threshold used was 80,000. A 488-nm wavelength argon laser was used to excite the cells (autofluorescence) and dye them. CFDA fluorescence was measured on the FL1-H long pass filter (533/530 nm) and side-scatter light (SSC)/fluorescence intensity data were analyzed. Daily sampling of all the samples was carried out.

### 2.4. Analytical Methods

#### 2.4.1. Enological Analysis

Samples were centrifugated at 28,000× *g* for 5 min at 4 °C. Sugar concentration and ethanol degree were monitored daily by FTIR (Fourier-transformed infrared) spectroscopy (OenoFOSS^TM^, FOOS, Hilleroed, Denmark). Daily sampling of all the samples was carried out.

#### 2.4.2. Volatilome Analysis

Headspace solid-phase microextraction gas chromatography/mass spectrometry, as reported previously [23,24], was used to quantify volatile compounds at the end of alcoholic fermentation for three of four biological replicates. In brief, 2 mL of sample was placed in a 10 mL vial with a silicone septum and then placed in an oil bath at 40 °C with a magnetic stirrer (700 rpm) for 10 min. A divinylbenzene/carboxene/polydimethylsiloxane (DVB/CAR/PDMS) fiber (Supelco, Bellefonte, PA, USA) was exposed to the sample headspace for 30 min and then subjected to direct desorption in the injector of the gas chromatograph set at 260 °C. Volatile compounds were analyzed by gas chromatography coupled to a quadrupolar mass-selective spectrometer. GC–MS analysis was performed in complete scanning mode (SCAN) in the 30–300 mass-unit range. The comparison of mass spectra and retention times with standard compounds allowed for the identification of compounds. In a few cases, a tentative identification was carried out based on the mass spectra using Wiley’s library 6-reference spectral databank. The quantitative assessment of volatiles was based on calibration curves obtained analyzing different concentrations of reference compounds in 10% hydroethanolic solution. The results were processed by performing an ANOVA (*p*-value < 0.01), followed by a Tukey test. All the results were processed using R software (R-4.0.4).

#### 2.4.3. Non-Volatile Metabolome Analysis

Ultra-high-resolution mass spectra were acquired using an FT-ICR-MS (SolariX, Bruker Daltonik, Bremen, Germany) equipped with a 12 Tesla superconducting magnet (Magnex Scientific Inc., Kidlington, UK) and an Apollo II electrospray ionization source (Bruker Daltonik, Bremen, Germany) operated in negative ionization mode. Samples were collected at the end of alcoholic fermentation and diluted at 5:100 *v*/*v* in pure methanol (LC-MS grade, Fluka, Germany). Quality controls (QC) were prepared by pooling equal amounts of all samples. QCs were analyzed at the beginning and end of the run and every ten samples to validate the repeatability of the measurement. The diluted samples and QC samples were injected at a flow rate of 120 µL/h into the electrospray ion source. Spectra were acquired with a time domain of 4 megawords within a mass range of m/z 147–2000. At total of 300 scans was accumulated for each sample. All the samples were injected randomly in the same batch to avoid batch variability. A resolving power of 400,000 at 300 m/z was achieved. All the spectra were calibrated externally on a methanol solution of arginine clusters (10 mg/L), and accuracy reached values of less than 0.1 ppm in day-to-day measurements. Additionally, internal calibrations were performed on a list composed by recurrent compounds in wine to keep only m/z peaks with a signal-to-noise (S/N) ratio of 4. Matrix Generator software (v. 0.4, Helmholtz-Zentrum Muenchen) was used to align each peak with a mass-accuracy window of 1 ppm. The quality of the analysis was controlled before any data processing (Supplementary Figure S1). Features were annotated using the in-house software NetCalc 2015 (v.1.1a, Helmholtz-Zentrum Muenchen). Finally, van Krevelen diagrams were generated by an Excel file according to the H/C versus O/C ratio of annotated metabolites. Annotation levels were in line with Viant et al., 2017 [25]. Perseus 1.5.1.6 (Max Planck Institute of Biochemistry, Germany) was used to perform principal-component analysis (PCA), hierarchical cluster analysis (HCA), and analysis of variance (ANOVA). For HCA, the Euclidean distance and average linkage were chosen, and a threshold *p*-value of 0.05 was chosen for ANOVA. Multidimensional Stoichiometric Compounds Classification (MSCC) have been used to elucidate extracted biomarkers categories commonly defined as lipids, peptides, amino sugars, carbohydrates and polyphenols derivatives compounds [26]. 

Metabolites were identified by ultra-high-performance liquid chromatography (Dionex Ultimate 3000, ThermoFischer, Waltham, MA, USA) coupled to a MaXis plus MQ ESI-Q-ToF mass spectrometer (Bruker, Bremen, Germany). A reversed-phase liquid-chromatography (RP-LC) separation method was applied by injecting 5 μL in an Acquity UPLC BEH C18 1.7 μm column 100 × 2.1 mm (Waters, Guyancourt, France) to separate metabolites according to their polarity. Buffer A (acetonitrile 5% (*v*/*v*) with 0.1% (*v*/*v*) formic acid) and buffer B (acetonitrile with 0.1% (*v*/*v*) formic acid) were employed to elute metabolites. Detection was implemented in negative ionization mode with the following parameters: an electrospray (Nebulizer pressure = 2 bars and nitrogen dry gas flow = 10 L/min), ion transfer (end plate offset at 500 V) capillary voltage (at 4500 V), and acquisition (100–1500 m/z mass range).

A mix of standard peptides and polyphenols was used for the UHPLC-Q-ToF-MS quality control. Experimental quality control (mix of samples) was used to guarantee system repeatability. All the samples were injected randomly in the same batch to avoid batch-to-batch variability. Calibration was processed with ¼ diluted ESI Tuning Mix (v. 4.3, Bruker Daltonik GmbH). Features (couple of m/z-values and retention times) were fragmented using the AutoMS/MS function on the most intense features, with a frequency of 2 Hz. Fragmentation was done at three different collision energies: 15, 25, and 35 eV. After acquisition, MS/MS spectra were manually extracted using Bruker Data Analysis 4.4 (Bruker Daltonic, Bremen, Germany).

### 2.5. Descriptive Sensory Analysis

In order to have enough volume per sample to carry out descriptive sensory analysis, the four biological replicates of each yeast modality were pooled. The twelve post-alcoholic fermentation wines were then filtered and sulfited to 40 mg/L before bottling. These samples were stored in the cellar at a constant temperature for one month until sensory analysis.

The assessors were recruited from among the enology students at the Dijon School of Enology at the Institut Universitaire de la Vigne et du Vin. They all attended 56 h of training in winetasting. The 33 candidates were subjected to a selection process during which their ability to identify the main white wine attributes and their reproducibility was checked. The final descriptive panel was composed of 22 assessors (14 males, 8 females, average age of 26).

The final sensory measure was carried out in a single session divided into two series of twelve samples (about 45 min per series). The session was conducted in a sensory evaluation room with individual boxes. Each sample was coded by a three-digit random number. Thirty mL of each wine was served at room temperature in standard ISO black glasses and covered with a plastic Petri dish. The samples were assessed in a specific order for each panelist following a Latin square. Samples were described according to the frequency-of-citation method [27]. The panel was asked to describe the odor of each sample by checking the associated descriptors within a pre-established list of 33 terms corresponding to odor attributes related to white wines. The list was adapted from previous research [28] and is presented in Supplementary Material, Table S1. Two blank lines were added to the list in order to allow the participants to use descriptors that were missing from the list, so they could add their own descriptors. For each term, the frequency of citation was established for each wine. Only the descriptors mentioned at least 4 times for at least one wine were kept for statistical analysis. The other descriptors were removed. The sensory data were subjected to correspondence analysis (CA) (α = 0.05), followed by hierarchical cluster analysis on the wine coordinates of the most relevant CA factors. Sensory statistical analyses were performed using R software (R-4.0.4).

## 3. Results and Discussion

Twelve *Saccharomyces cerevisiae* strains were screened. All these strains were selected for their phenotypic diversity, diverse origins, and genetic backgrounds. The twelve strains were commercial stains widely used in the wine industry. For example, S2 was obtained by an adaptative evolution from strain S1 [29]. Strain S7 was a natural hybrid of strain S10 that had been crossed with another *Saccharomyces cerevisiae* strain, and S6 was a hybrid obtained by UV mutagenesis [30]. All the strains exhibited different nitrogen requirements [31,32], which can be associated with different profiles of aromatic compound formation [33].

### 3.1. Fermentation Kinetics and Yeast Viability

Alcoholic fermentation of Chardonnay must inoculated by 12 different yeast strains was monitored by IRTF spectroscopy (OenoFoss) and flow cytometry every day until the end of fermentation. All twelve strains showed similar growth and fermentation kinetics or patterns. The results of some fermentation parameters are expressed in the Supplementary Data (Table S2). The strains all showed a rapid exponential phase (48 h). No residual sugars remained at the end of the monitoring period, except for strain S8 (Supplementary Table S2). This last yeast reached dryness at 264 h. The maximum population was within the range of 7.95 × 10^7^ to 1.46 × 10^8^ viable cells/mL. The ethanol yield was whatever the strains, which was in accordance with previous studies [34,35,36]. S12 consumed 20% of initial malic acid, which had already been described earlier [37]. Comparable kinetics were observed between the strains, which was expected since all the strains belonged to the same species and were selected for their alcoholic fermentation performances [38,39].

### 3.2. Specific Footpinting of Strains

In our experiment, the twelve strains fermented the same must; thus, in these conditions, our metabolomic analysis reflected the specific traits of each strain. Indeed, statistical analysis of our data revealed a unique footprint for each of the strains studied.

**Figure 1:**
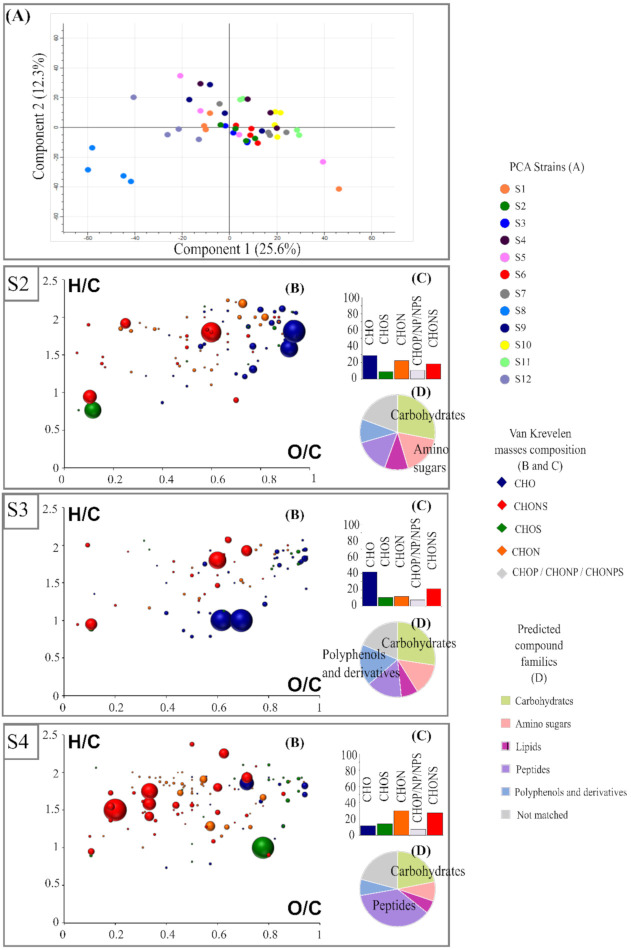
(**A**) Principal-component analysis of twelve *Saccharomyces cerevisiae* strains based on FT-ICR-MS data using direct methanol dilution. ANOVA (*p* < 0.05) was used to extract specific markers for each of the twelve strains: S2, S3 and S4 are represented as examples. For each strain, H/C vs. O/C van Krevelen diagrams (**B**), histogram proportions that show their elemental compositions (**C**), and a pie chart (**D**) representing the distribution of these markers by hypothetical families of common wine compounds are presented. Bubble sizes indicate relative intensities of corresponding masses. Color code: CHO, blue; CHON, orange; CHONS, red; CHOS, green.

A principal-component analysis (PCA) was performed on the FT-ICR-MS data to display the differences in the metabolomic composition of wine by representing the biological replicates of each strain. The PCA score plot of the two first components (Figure 1A) explained 37.9% of the metabolic variation. This representation highlighted the great proximity of the replicates for a given strain. At the strain level, this projection on these two axes allowed for separation of strains based on the metabolite produced and revealed high metabolic diversity. Our non-targeted analysis allowed us to report, for the first time, considerable metabolic differences between strains within the same species.

The first principal component accounted for 25.6% of the variation and enabled the clear differentiation of the related strain as S1 and its adaptative evolution as S2. This first study of the exometabolome of twelve strains of the same species under fermentative conditions also reflected metabolically different strains that are associated with phenotypic differences. For example, the relevant separation, according to axis 1 of strain S8 to the other strains, may be related to the sluggish fermentation described earlier. A significant change in the exometabolome seemed to contribute to this phenomenon, consistent with the impact of various known metabolites already described [40,41].

A total of 58,392 masses detected in FT-ICR-MS was extracted. Of these, the unique masses were excluded for further data processing. As a result, 2685 masses (Level 4) were retained. Then, among these masses, 2179 could be associated with a molecular formula (Level 3). Among them, it was possible to distinguish molecular formulas that were unique to a strain from a molecular formula whose abundance varied significantly, depending on the strain. Molecular formulas that presented significant differences in their mean intensity between all twelve strains were extracted by performing ANOVA statistical analyses (*p*-value < 0.05) and were considered as biomarkers. For each of these, their mass was subjected to different databases (KEGG, MassTrix, Metlin, YMDB) for putative annotation (Level 2).

Despite a similar metabolic background shown in the representation of common masses (Supplementary Figure S2), the strains were differentiated from each other by a pool of specific compounds extracted statistically (Table 1). Out of the 2179 molecular formulas, 1380 were common to all strains. Thus, 36.7% of the detected non-volatile composition was different, depending on the strain. As stated above, this proximity could be explained by the fact that all the strains belong to the same species and have very close genetic backgrounds [42] S8, which stood out with PCA, unsurprisingly had the highest number of specific markers (1162) (Table 1).

Looking further ahead, it was very interesting to know the nature of the differences in chemical composition of biomarkers for each strain. The set of biomarkers for a given strain matched their metabolomic footprint, which was previewed by van Krevelen diagrams. The van Krevelen diagrams were based on their O/C and H/C ratios (Figure 1). The wide range of biomarkers of each strain and its composition highlighted the diversity of metabolisms among these strains. Differences in both quantitative and chemical composition could be observed. This metabolic diversity was also illustrated by associated histograms of proportion-marker composition for each strain (Figure 1, Supplementary Figure S3). Thus, S3 was predominantly associated with CHO markers in the area of the van Krevelen diagram where carbohydrate- and polyphenol-derivative compounds were expected. On the contrary, the S4 profile appeared distinctly different from the other strains, with markers mainly composed of CHON and CHONS. The latter were located in the area associated with peptides on the van Krevelen diagram and whose composition is in agreement with Rivas-Ubach et al., 2018 [26]. S5 and S7 were characterized by a large number of CHO, closely followed by CHON, and finally, CHONS.

**Figure 2:**
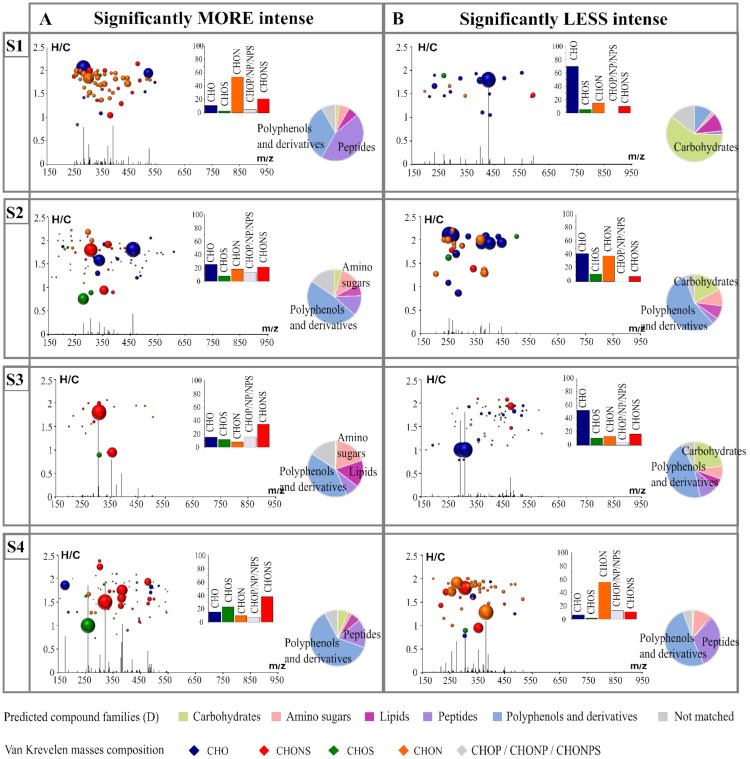
H/C vs. m/z van Krevelen diagrams combined with intensity vs. m/z diagrams coupled to histogram proportions of the elemental formula compositions exhibit specific strain markers significantly less (**B**) and more (**A**) intense in each fermentation. Bubble sizes indicate relative intensities of corresponding masses. Color code: CHO, blue; CHON, orange; CHONS, red; CHOS, green. The pie chart represents the distribution of these markers by hypothetical families of common wine compounds.

Furthermore, to determine the impact of each strain on their biomarkers, we extracted biomarkers that were significantly more intense (Figure 2A) and less intense (Figure 2B) for each strain. The histogram proportions show the differences in qualitative elemental composition, and the pie charts support the prediction of the families of compounds that can be attributed to each feature. A diagram representing the H/C ratio as a function of the m/z ratio combined with the intensity of each of the features according to their m/z was used. This alternative representation provided additional information on the molecular weight of the biomarkers. All the features were in the mass range of 150 to 650 m/z. We found that the features extracted showed a large variability in composition between those that were less and more intense (Figure 2 and Supplementary Figure S4). For example, we observed that for strain S1, the significantly more intense markers were mainly composed of nitrogen-containing compounds (potential peptides and polyphenol-derivative compounds), while the less intense ones were mostly associated with CHO markers in the area of the van Krevelen diagrams where carbohydrate compounds were expected. Biomarkers of S2, whether they were more or less intense, presented peaks with lower intensity in comparison with the other strains.

Once the markers were extracted and their nature assumed, it was possible to assign a hypothetical annotation (Level 3) to each of them using databases (KEGG, YMDB, Metlin, Lipidmap, Oligonet). In our case, only 10 to 15% of the specific markers could be annotated in the databases, confirming previous conclusions that evoked the complexity of wine composition and the lack of knowledge on it in the current databases [3,17,43]. This step also allowed for the determination of the metabolic pathways associated with the metabolic modifications expressed by the specificity of the markers of each strain (Supplementary Figure S5). Thirty-two metabolic pathways were involved in exometabolome changes and include from 1 to 105 biomarkers. Among the most represented pathway, central metabolic pathways were found, such as carbon metabolism (67 biomarkers) or pyruvate metabolism (67 biomarkers). Moreover, there were also metabolic pathways involved in the synthesis of amino acids, such as phenylalanine (94 biomarkers). This last pathway is one of the many examples highlighting the importance of nitrogen metabolism, which appeared to be the most affected pathway. Indeed, most of the strain differentiation was associated with nitrogen-containing compounds. The significantly less intense biomarkers associated with the S2 strain, an adaptive evolution of the S1 strain, were predominantly nitrogen compounds, unlike S1. S1 was previously described by overexpression of genes coding amino-acid metabolism [31]. The adaptive evolution leading to the stimulation of the pentose phosphate pathway would therefore have fewer intermediate metabolites. This was confirmed by the absence of intermediate biomarkers of this metabolic pathway.

To confirm the structure of the previously annotated compounds, we performed an LC-MS/MS analysis and a comparison with the METLIN fragmentation database. A total of eight structures was confirmed (Level 2) (Supplementary Table S3) among the annotated masses.

The power of this approach made it possible to extract metabolites according to the strains used to conduct alcoholic fermentation. Thus, it was possible to determine the unique impact of each strain on the different types of metabolites and the related metabolic pathways. This also revealed intraspecific diversity through the non-volatile exometabolome. The non-targeted metabolomic approaches in the literature are mainly focused on secondary metabolism and therefore on the volatile exometabolome [43]. To our knowledge, this is the first time that a non-targeted approach focused on the main metabolism was conducted on a large panel of strains used in the wine industry.

Metabolic diversity was mainly observed at the level of nitrogenous metabolism, which plays a major role in the synthesis of volatile compounds. Therefore, it seemed important to investigate the intraspecific diversity on the secondary metabolism scale.

### 3.3. Impact of Strains on the Volatilome

**Figure 3:**
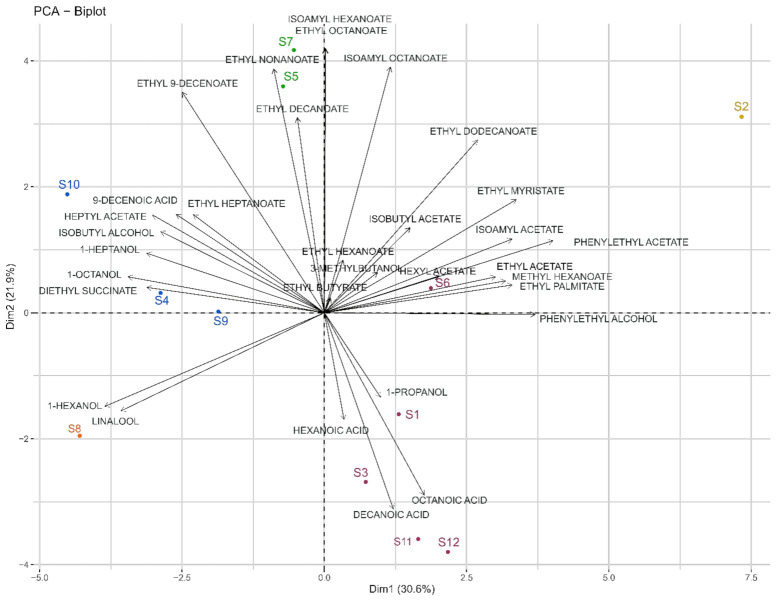
Biplot of PCA (Dim1 vs. Dim2) analysis applied to significantly different volatile compounds found in the twelve fermentations carried out by the different strains of *Saccharomyces cerevisiae*. Colored samples represent different classes obtained from HCA.

HS-SPME-GC/MS was used to dose 35 volatile compounds in our fermentations. The volatile composition of each sample is presented in detail in Supplementary Table S4. A one-way ANOVA with strain as main effect was performed for each compound. The results showed that 31 compounds out of 35 showed significant differences between yeast strains. The four compounds showing non-significant differences were nonanol, benzyl alcohol, nonanoic acid, and benzaldehyde. For our study, only the discriminant compounds were considered for further statistical analyses. The ester group was most represented, with 20 esters identified and quantified. PCA and HCA were applied to volatilome data and allowed for the differentiation of five groups of strains based on Euclidean distance and the Ward method (Figure 3).

The PCA score plot of the first two components explained 52.5% of the variation (Figure 3), with the first principal component (Dim1) accounting for 30.6% and the second (Dim2) accounting for 21.9%. The five clusters of samples from the HCA were projected onto this PCA using different colors.

The S8 and S2 strains appeared to form separate groups. The sluggish fermentation of S8 observed was also associated with a distinct volatilome and was in line with a contrasting metabolomic signature. This strain correlated with 1-hexanol and linalool. S2 is associated with isoamyl acetate, ethyl myristate, and phenylethyl acetate. On the other hand, other groups, like S5 and S7, were related to ethyl nonanoate and decanoate, while S1, S3, S6, and S12 were associated with three medium-chain fatty acids (MCFA) (hexanoic, octanoic, and decanoic acids). Finally, S4, S9, and S10 were correlated with higher alcohols, such as 1-octanol and 1-heptanol.

**Figure 4:**
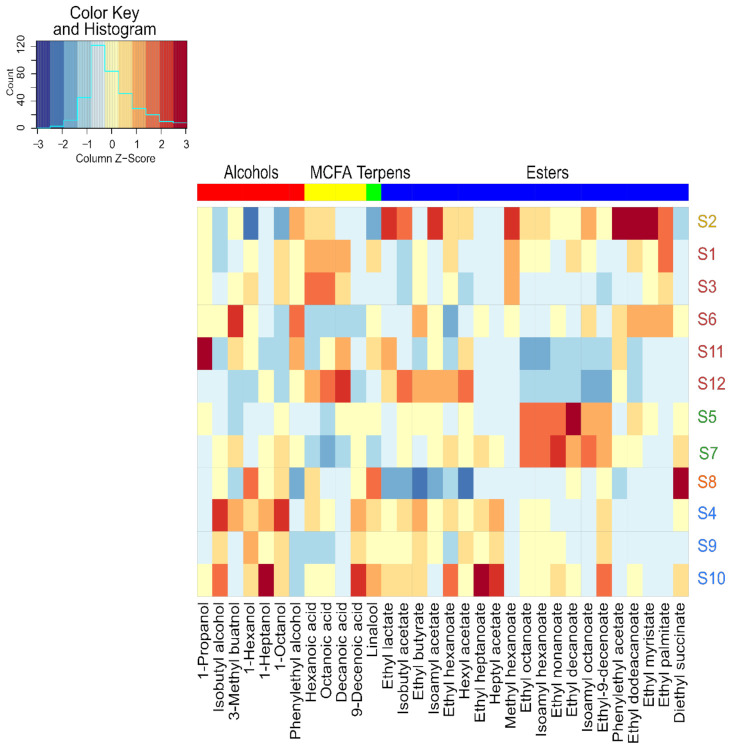
Heat map of volatile compounds produced by the different strains of *Saccharomyces cerevisiae*. Columns correspond to volatile compounds that presented significant differences between twelve strains. Colored samples represent different class obtained from HCA.

Another representation made it possible to highlight that across our twelve strains, an overexpression of certain families of VOCs by some strains (Figure 4), in addition to the significant diversity of expression of volatile compounds, was observed. S5 and S7 overexpressed the same six esters, and S4 overexpressed mostly higher alcohols. S2 resulted from an adaptative evolution by orientation of the carbon flow towards the pentose phosphate pathway, overexpressing 8 of the 20 quantified esters, confirming previous studies [44,45].

On the contrary, parental strain S1 predominantly overexpressed intermediates of these esters as MCFA. Indeed, Cadière et al., 2012 [44] reported an increase in the concentration of some acetate esters by strain S2 evolved from parental strain S1. For example, the concentration was 2.5, 3.2, and 3.2 times higher for isoamyl acetate, isobutyl acetate, and phenylethyl acetate, respectively.

The initial composition of the matrix in nitrogenous sources [46], as well as the preferential nitrogenous sources [47], could lead to this modulation of the concentration of the volatile compounds according to the strain of *Saccharomyces cerevisiae* involved. Furthermore, this variety of production of these compounds is subject to the expression of several genes and variations in the expression level of several transcription factors [48,49,50]. Eder et al., 2020 [51] also recently highlighted the involvement of loci and genes in nitrogen metabolism and its assimilation, influencing the formation of these volatile compounds, using the QTL mapping approach. This confirms the key role of nitrogen metabolism, the expression of related genes, and their regulation in the diversity of *Saccharomyces cerevisiae* strains at different scales. Our exometabolomic study underlined those differences in nitrogen metabolism and explained differences between strains, which supports previous findings using molecular approaches.

As described above, our results showed that it is possible to discriminate samples, thereby confirming intraspecific diversity in *Saccharomyces cerevisiae* species [43] and the considerable metabolic diversity of our strains. The latter reflected the intraspecific metabolic diversity observed during the study of the non-volatile exometabolome. Indeed, primary metabolism participates in the expression of secondary metabolism and thus in its diversity. However, these two approaches remain complementary. Chemical diversity was therefore observed from another angle. Indeed, FT-ICR-MS targeted a range of analyzed masses corresponding to the non-volatile metabolome of yeast. Furthermore, 35 volatile compounds were targeted for quantification, but this is not fully representative of the diversity of non-volatile compounds present in wines. Therefore, sensory analysis is still a valuable means of obtaining a comprehensive view of this intraspecific diversity.

### 3.4. Sensory Impact of Intraspecific Diversity

At the end of the alcoholic fermentation, the wines from the organic replicates of each yeast modality were pooled, sulfited, filtered, bottled, and stored for one month. Frequency citations of each attribute were computed for each sample.

**Figure 5:**
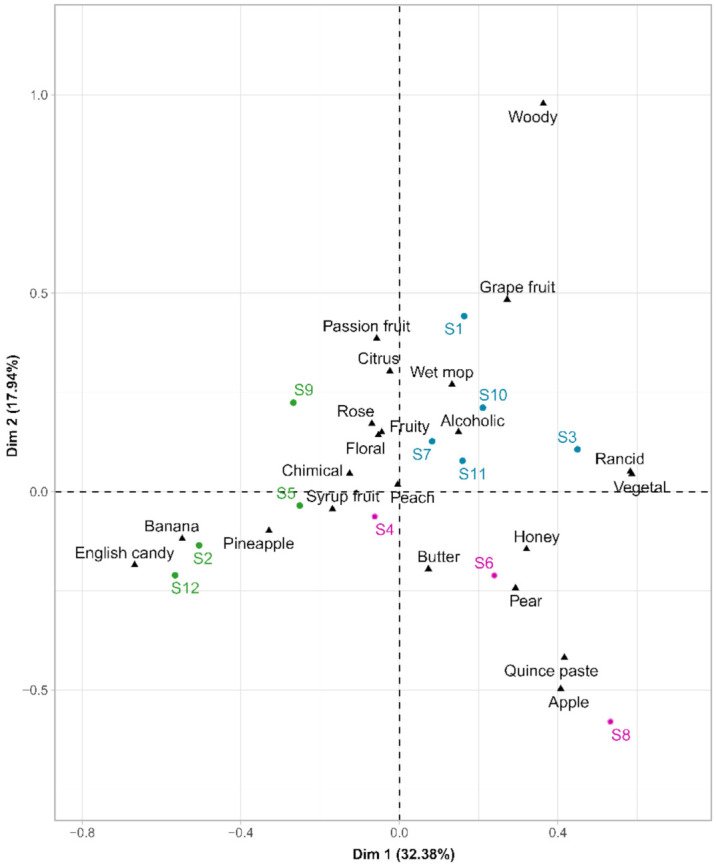
Biplot of the correspondence analysis of the general description of the twelve wines. Colors indicate the clusters obtained by HCA (using Ward’s criterion) on the two first CA dimensions.

The resulting contingency matrix was subjected to a correspondence analysis (CA), shown in Figure 5. The first two principal components of this CA, Dim1 and Dim2, explained 50.32% of the variance. The distribution of wine samples displayed a considerable separation between wines made with distinct yeast strains. Hierarchical cluster analysis (HCA) was carried out on wine coordinates on the two first dimensions of the CA. The samples were separated into three main groups, represented in various colors (Figure 5).

The first group (in blue), located in the positive values of the first dimension, included samples S1, S7, S10, S11, and S3, which can be described by the attributes: “grapefruit”, “wet mop”, “rancid”, “vegetal”. The second cluster (in green) was composed of wines from fermentations conducted with the S5, S9, S2, and S12 strains and were located on the negative side of the first dimension. Within this cluster, S2 and S12 were characterized as “banana” and “English candy”, while S5 and S9 were “chemical” and “fruity”. The last group (in pink), composed of strains S4, S6, and S8, was characterized by oxidation terms as “quince paste”, “honey”, “butter”. Our results showed quite distinctive aroma profiles among the strains studied, demonstrating intraspecific diversity at the sensory level [52,53]. It was even possible to discriminate finished wines fermented by certain related strains. The S2 strain, resulting from the evolutionary adaptation of the S1 strain, belonged to different sensory classes, and hence, led to distinct sensorial profiles. Nevertheless, this was not the case for the S7 hybrid and the parental S10 strains. Despite the significant role of volatile compounds, their presence could not simply explain the sensory differences [54,55]. Moreover, despite a complex matrix, interactions between volatile compounds, and important differences in the perception threshold between the different aromatic volatile compounds, some descriptors could be related to volatile organic compounds. A high concentration of isoamyl acetate for strains S2 and S12 was significantly associated with very fruity terms, including “banana” and “English candy”, as mentioned in previous studies [44,56]. Concerning the vegetal term, we found a significant correlation with 1-hexanol, 1-heptanol, which has also been reported by other authors [57,58]. Moreover, 1-propanol was significantly linked to the term “rancid”, which had already been related to pungent flavors [20].

As for the two other approaches, it was possible to distinguish the finished wines produced thanks to the different strains of *Saccharomyces cerevisiae* used. The metabolic differences were, in fact, expressed in distinct phenotypes with regard to volatilome and sensory properties. We could see that the groups established based on the volatile composition analysis of the metabolome did not correspond exactly to the classes established from the sensory analysis. Furthermore, we observed that only a few sensory descriptors could be correlated to volatile compounds, as previously stated. Thus, this confirms previous studies, which revealed the difficulty of correlating the sensory aspect and the dosage of volatile compounds.

Synergetic phenomena were observed between volatile compounds [59]. Conversely, the masking of a volatile compound by another was demonstrated [57,60,61]. Thus, the interactions between volatile compounds lead to diverse aromatic profiles. Moreover, in this study, the dosage of volatile compounds remained non-exhaustive and therefore not representative of the aromatic profile of wine at the sensory level.

To our knowledge, this was the first time that these three complementary approaches were combined for a comparative study.

## 4. Conclusions

This work reported the metabolic diversity within the *Saccharomyces cerevisiae* species under fermentative conditions from different perspectives. Ultra-high-resolution mass spectrometry (uHRMS) proved to be a powerful tool for the discrimination of yeast strains of the same species—in this case, *Saccharomyces cerevisiae.* A wide range of non-volatile metabolites belonging to different families of compounds, such as carbohydrates, peptides, lipids, aminosugars, polyphenols, and derivatives, was detected. Each of the strains could be associated with a specific metabolomic fingerprint, including unique markers and biomarkers that varied significantly in intensity. Thus, using this technique, we were able to highlight metabolic differences between strains of the same species that presented the same technological performances. Furthermore, the significant involvement of nitrogen metabolism in this differentiation was considered. It was also possible to show that the modulation of metabolism observed at the level of the non-volatile exometabolome was observed from the perspective of the volatilome and sensory aspect. Therefore, phenotypic differences within the same species revealed metabolic differences that resulted in the diversity of the volatile fraction that participates in the palette of the sensory pattern.

UHRMS analysis may be used for discriminating strains within yeast species after the alcoholic fermentation process and used in combination with other approaches to establish an integrative view of intraspecific diversity.

## Figures and Tables

**Table 1:** Table of extracted, annotated, and identified metabolites according to wine fermented by each strain of *Saccharomyces cerevisiae* (S1 to S12).

Strains	Number of Extracted Masses	Number of Biomarkers (Level 4)	Unique Molecular Formulas	Tentative Structure (Level 3)	Fragmented Biomarkers (Putative Identification Level 2)	Validated Identification (Level 2)
S1	1513	133	0	6	1	0
S2	1588	208	0	16	5	1
S3	1507	127	0	8	4	2
S4	1669	289	0	15	3	0
S5	1508	128	0	5	0	0
S6	1545	165	0	7	5	0
S7	1510	130	0	7	1	0
S8	2576	1162	34	37	10	4
S9	1602	222	0	17	3	1
S10	1782	402	0	21	2	0
S11	1689	309	0	15	0	0
S12	2051	668	3	32	0	0

## Supplementary Materials

The following are available online at https://www.mdpi.com/article/10.3390/microorganisms9112327/s1, Figure S1: (A) Time series plot of the first principal component (t(1) vs. sample run order) (B) Plot representing the maximum, mean, and minimum peak intensity for each of the 9 QCs. (C) Histogram of all coefficients of variation values computed from the peak intensities from all the detected molecular compositions in the FT-ICR-MS data. (D) Extracted ion chromatograms of m/z 149.00917 in 9 sequential QCs samples., Table S1: Ballot used for the general odor description (translated from French), Table S2: Cell viability and fermentation parameters for the 12 strains of *Saccharomyces cerevisiae* (S1 to S12), Figure S2: Compounds common to all samples detected after methanol dilution. H/C vs. O/C van Krevelen diagrams, histogram proportion showing their elemental compositions; H/C vs. m/z van Krevelen diagrams combined with intensity vs. m/z diagrams coupled to histograms proportions of the elemental formula compositions, and pie chart representing the distribution of these markers by hypothetical families of common wine compounds adapted from Rivas and Ubach et al., 2018 [23]. Bubble sizes indicate relative intensities of corresponding masses. Color code: CHO, blue; CHON, orange; CHONS, red; CHOS, green. Figure S3: Foot printing of twelve *Saccharomyces cerevisiae* strains based on FT-ICR-MS data using direct methanol dilution. ANOVA (*p* < 0.05) was used to extract specific markers for each of the twelve strains. For each strain, H/C vs. O/C van Krevelen diagrams (B), histogram proportion (C) that show their elemental compositions and pie chart (D) representing the distribution of these markers by hypothetical families of common wine compounds adapted from Rivas and Ubach et al., 2018 [23]. Bubble sizes indicate relative intensities of corresponding masses. Color code: CHO, blue; CHON, orange; CHONS, red; CHOS, green., Figure S4: H/C vs. m/z van Krevelen diagrams combined with intensity vs. m/z diagrams coupled to histograms proportion of the elemental formula compositions exhibits specific strains markers significantly less and more intense in each fermentation. Bubble sizes indicate relative intensities of corresponding masses. Color code: CHO, blue; CHON, orange; CHONS, red; CHOS, green. The pie chart represents the distribution of these markers by hypothetical families of common wine compounds adapted from Rivas and Ubach et al., 2018 [23], Figure S5: Number of biomarkers that could be associated with the different metabolic pathways of *Saccharomyces cerevisiae* using MassTrix and Kegg, Table S3: Table of extracted, annotated, and identified metabolites according to wine fermented by each strain of *Saccharomyces cerevisiae* (S1 to S12). Levels of annotations were derived from Viant et al., 2017 [26], Table S4: Table of extracted, annotated, and identified metabolites according to wine fermented by each strain of *Saccharomyces cerevisiae* (S1 to S12). Levels of annotations were derived from Viant et al., 2017 [26].
