# Supplementary material for: Different Wines from Different Yeasts? “Saccharomyces cerevisiae Intraspecies Differentiation by Metabolomic Signature and Sensory Patterns in Wine”
Source: Microorganisms. 2021 Nov 10;9(11):2327. doi: 10.3390/microorganisms9112327 (PMC8620830; doi:10.3390/microorganisms9112327)
Supplement: Supplementary file 1 [file microorganisms-09-02327-s001.zip › microorganisms-1408782 supplementary org.pdf]

Supplementary data

Different wines from different yeasts? "*Saccharomyces cerevisiae* intraspecies differentiation by metabolomic signature and sensory patterns in wine"

Fanny Bordet [1,5*], Chloé Roullier-Gall [1], Jordi Ballester[3], Stefania Vichi[4], Beatriz Quintanilla-Casas[4], Régis D.Gougeon[1,2], Anne Julien-Ortiz[5], Philippe Schmitt Kopplin[6] and Hervé Alexandre [1]

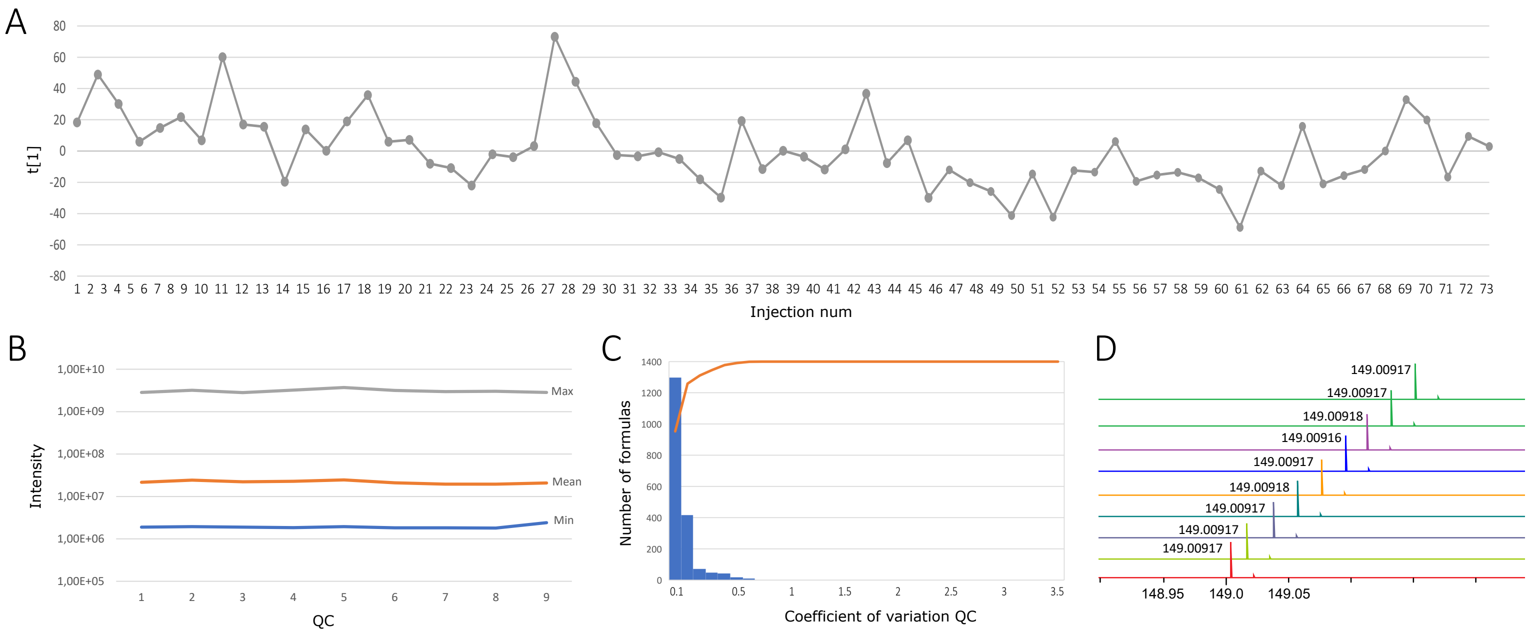

Figure S1 : (**A**) Time series plot of the first principal component (t(1) vs. sample run order) (**B**) Plot representing the maximum. mean and minimum peak intensity for each of the 9 QCs. (**C**) Histogram of all coefficient of variation values computed from the peak intensities from all the detected molecular compositions in the FT-ICR-MS data. (**D**) Extracted ion chromatograms of m/z 149.00917 in 9 sequential QCs samples.

Table S1 : Ballot used for the general odor description (translated from French)

Sample :

Smell (only with your nose) each of the wines presented in front of you. Remember to note the sample code on each of the cards.

Check the descriptors that **best** correspond to the smell of each sample:

☐ Earthy	☐ Vanilla	☐ Wet mop
☐ Red fruits	☐ Floral	☐ Chimical
☐ Apple	☐ Vegetal	☐ Green Pepper
☐ Honey	☐ Rotten egg	☐ Fruit in syrup
☐ Alcoholic	☐ Grape fruit	☐ White flower
☐ Woody	☐ Pear	☐ Quince paste
☐ Fruity	☐ Toasted	☐ Banana
☐ English candy	☐ Spicy	☐ ....
☐ Herbaceous	☐ Rancid	☐ ....
☐ Citrus	☐ Peach	☐ ....
☐ Rose	☐ Lemon	
☐ Petrol	☐ Pineapple	
☐ Butter	☐ Passion fruit	

Table S2 : Cell viability and fermentation parameters for the 12 strains of Saccharomyces cerevisiae (S1 to S12)

Strains	Maximum population (viable cells/mL)	Time todryness (hours)	Time to T50 (Hours)	Population decline before the end of fermentation	pH	Malic acid concentration (g/L)
**S1**	$1.06.10^{8}$	216	67.5	_	3.91	4
**S2**	$1.18.10^{8}$	168	60	YES (120 hours)	3.92	3.8
**S3**	$1.35.10^{8}$	216	67.5	_	3.89	3.9
**S4**	$1.37.10^{8}$	216	67.5	_	3.86	4
**S5**	$1.23.10^{8}$	216	70	_	3.86	3.9
**S6**	$1.16.10^{8}$	216	70	_	3.9	4.2
**S7**	$1.17.10^{8}$	168	60	_	3.84	4.1
**S8**	$1.28.10^{8}$	264	75	_	3.89	3.8
**S9**	$1.02.10^{8}$	216	75	_	3.86	3.5
**S10**	$7.95.10^{7}$	192	62.5	_	3.82	4.9
**S11**	$1.13.10^{8}$	192	67.5	_	3.79	3.9
**S12**	$1.46.10^{8}$	168	55	_	3.91	3.2

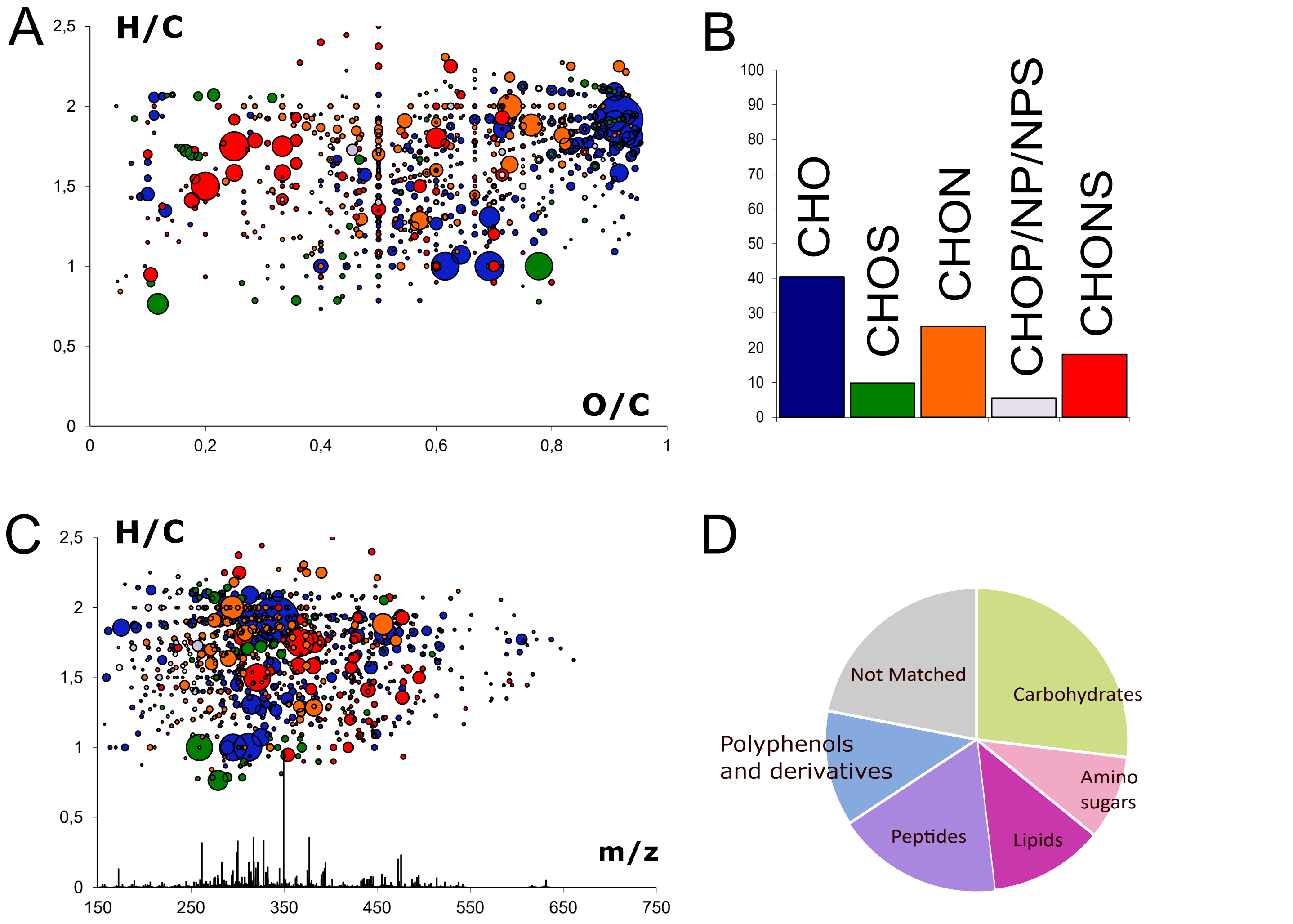

Figure S2 : Compounds common to all samples detected after methanol dilution. (**A**) H/C vs O/C Van Krevelen diagrams. histogram proportion showing their elemental compositions;. (**C**) H/C vs m/z Van Krevelen diagrams combined with intensity vs m/z diagrams coupled to histograms proportions (**B**) of the elemental formula compositions. and (**D**) pie chart representing the distribution of these markers by hypothetical families of common wine compounds adapted from Rivas and Ubach et al.. 2018 [23]. Bubble sizes indicate relative intensities of corresponding masses. Colour code: CHO. blue; CHON. orange; CHONS. red; CHOS. green.

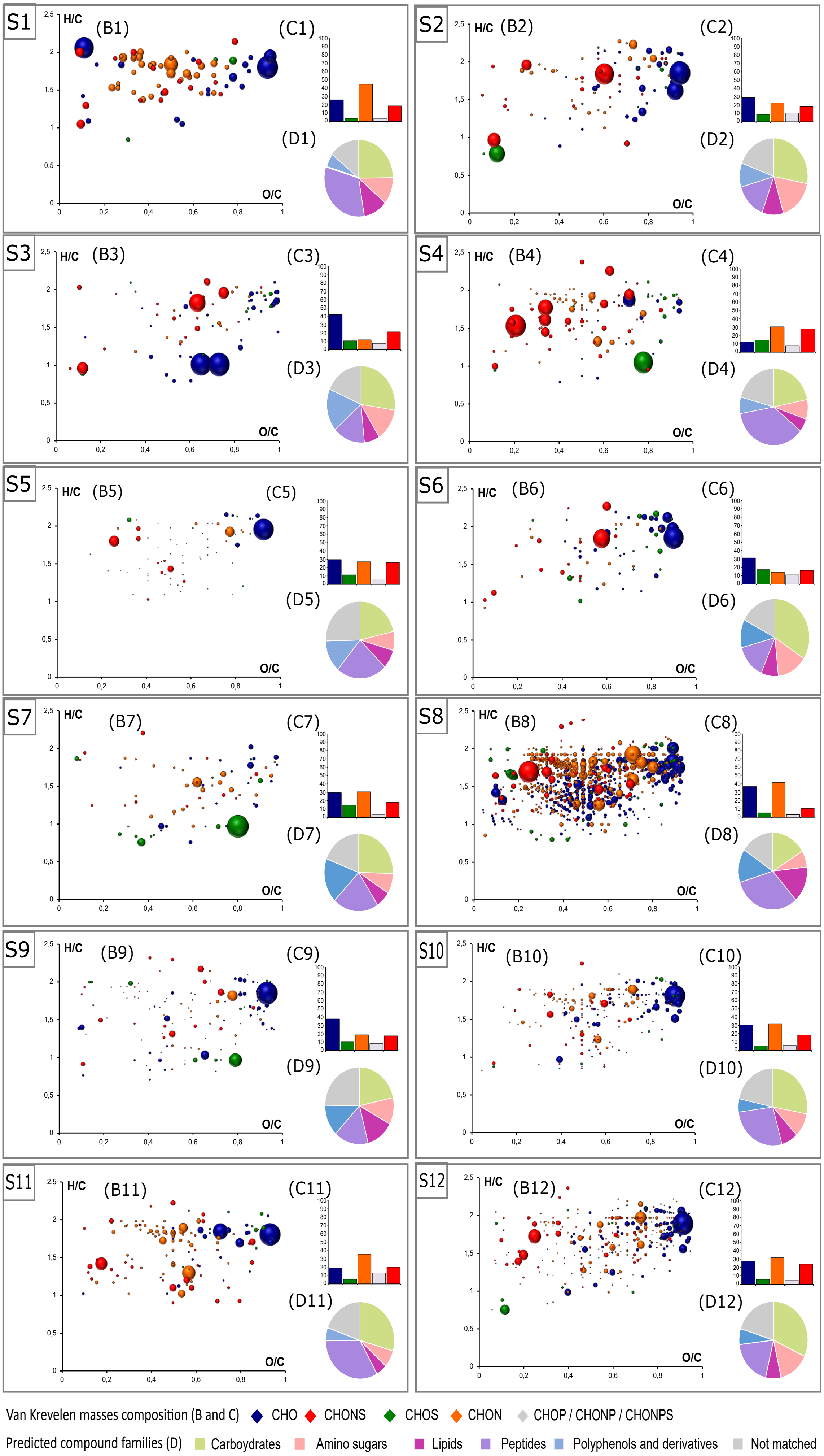

Figure S3 : Footprinting of twelve Saccharomyces cerevisiae strains based on FT-ICR-MS data using direct methanol dilution. ANOVA ($p<0.05$) was used to extract specific markers for each of the twelve strains. For each strain. H/C vs O/C Van Krevelen diagrams (B1–12). histogram proportion (C1–12) that show their elemental compositions and pie chart (D1–12) representing the distribution of these markers by hypothetical families of common wine compounds adapted from Rivas and Ubach et al.. 2018 [23]. Bubble sizes indicate relative intensities of corresponding masses. Color code: CHO. blue; CHON. orange; CHONS. red; CHOS. green.

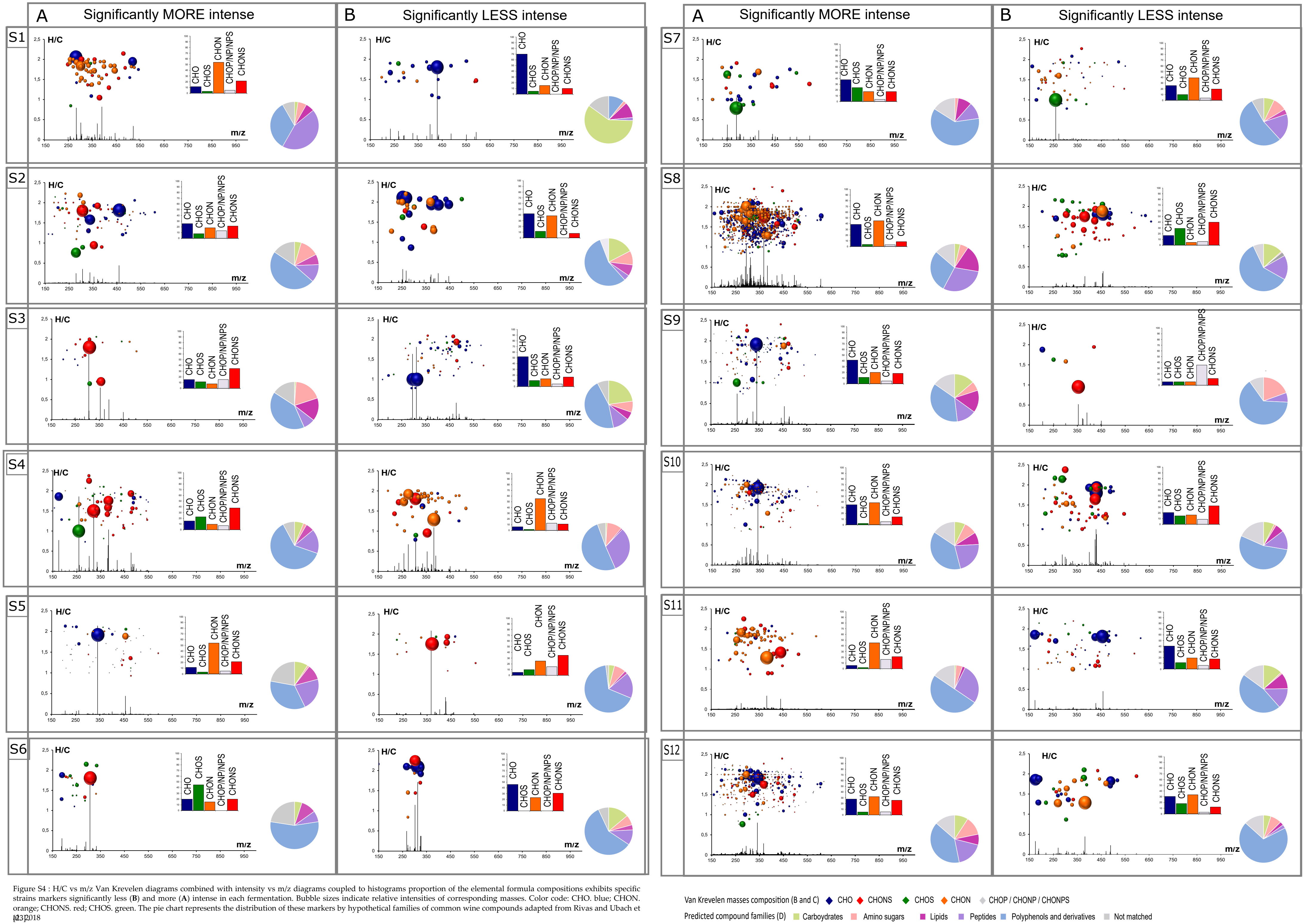

Figure S4 : H/C vs m/z Van Krevelen diagrams combined with intensity vs m/z diagrams coupled to histograms proportion of the elemental formula compositions exhibits specific strains markers significantly less (**B**) and more (**A**) intense in each fermentation. Bubble sizes indicate relative intensities of corresponding masses. Color code: CHO. blue; CHON. orange; CHONS. red; CHOS. green. The pie chart represents the distribution of these markers by hypothetical families of common wine compounds adapted from Rivas and Ubach et al. [13] 2018

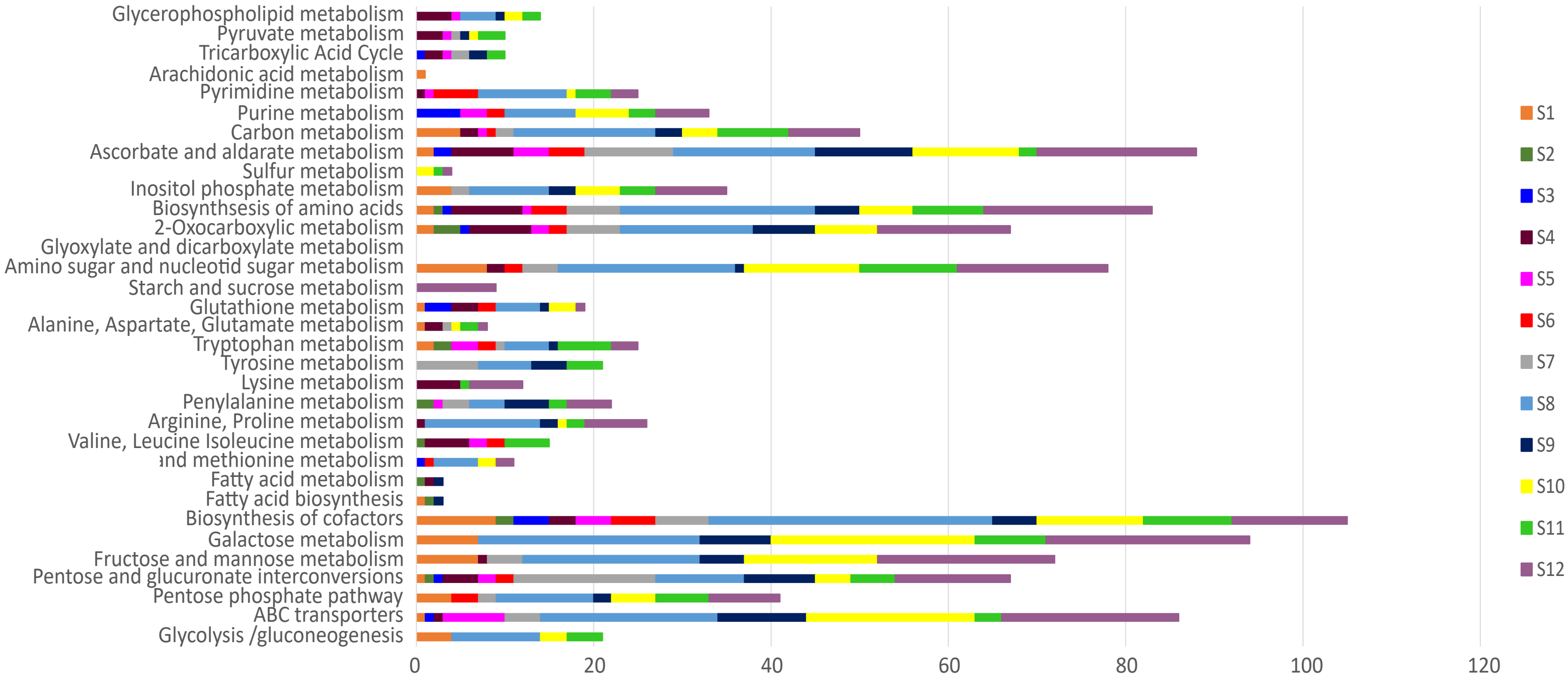

Figure S5 : Number of biomarkers that could be associated with the different metabolic pathways of Saccharomyces cerevisiae using MassTrix and Kegg

Table S3 : Table of extracted. annotated. and identified metabolites according to wine fermented by each strain of Saccharomyces cerevisiae (S1 to S12). Levels of annotations were derived from Viant et al.. 2017 [26]

	Biomarkers	Annotated biomarkers	Biomarkers fragmented by MS/MS	Biomarkers annotated fragmented by MS/MS
**S1**	133	6	1	0
**S2**	208	16	5	1
**S3**	127	8	4	2
**S4**	289	15	3	0
**S5**	128	5	0	0
**S6**	165	7	5	0
**S7**	130	7	1	0
**S8**	1162	37	10	4
**S9**	222	17	3	1
**S10**	402	21	2	0
**S11**	309	15	0	0
**S12**	668	32	0	0

Table S4 : Table of concentrations of each volatile compound with standard deviation and letter of significance

Volatile Compounds (µg.L -1)	Aroma descriptor	Significativity	S1	S2	S3	S4	S5	S6	S7	S8	S9	S10	S11	S12
ALCOHOLS														
1-PROPANOL	weak fusel	< 0.0001	10.6± 1.3bcd	11.7 ± 2.5bc	10.6 ± 1.5 bcd	7.9± 3.6 bcd	10.8± 1.7 bcd	9.4± 2.9bcd	7.3± 1.9 bcd	6.7± 1.3 cd	7.1 ± 2.6 cd	12.5 ± 3 b	33.9± 5.4 a	6.2 ± 1.5 d
ISOBUTYL ALCOHOL	apple	< 0.0001	13.5± 2.5 e	14.6± 3.7 de	15.7 ± 2.2 cde	28.5 ± 4.4 a	16.6± 1.3 cde	19.9± 3.9bcd	19.9± 2.3 bcd	17.7± 2.9 cde	21.1 ± 3.6bc	25.4 ± 4 ab	14.9± 3.5 de	17± 1.7 cde
3-METHYLBUTANOL		< 0.0001	180807.1 ± 5062.7 c	203599.4 ± 35780.8 bc	200161.8 ± 16725.8 bc	227513.4 ± 41555.5 ab	174482.5 ± 8972.2 c	257215 ± 30747.7 a	206620.7 ± 14528.4 bc	173838.6 ± 10610.1 c	189523.6 ± 25601.7 bc	191455.6 ± 16427.1 bc	207764.9 ± 45276.1 bc	175495 ± 26400.9 c
1-HEXANOL	green	< 0.0001	337.6± 19.7 bcd	205.7 ± 40.2 e	353.8 ± 23.7 abc	372.2± 53.3 ab	308.4± 15.8 cd	343.6 ± 57.2bc	298± 17.5 cd	409.6 ± 33.6 a	378.5± 54 ab	373.6 ± 36.2 ab	331± 48 bcd	279.3 ± 37.8 d
1-HEPTANOL	leafy	< 0.0001	198.9± 7.5 def	185 ± 25.3 ef	192.2 ± 12.3 def	431.6± 62.2 b	205.6± 21.2 def	204.8± 24.4def	293.1± 14.5 cde	327.1± 33.9 bc	306.1 ± 62.4 cd	689.7 ± 49.3 a	167.2 ± 23.3 f	285.8± 88.9 cde
1-OCTANOL	waxy	< 0.0001	48.7 ± 10.3 b	30.3 ± 9.6 d	50 ± 7.6 b	68.4 ± 12.3 a	44.9± 4.5bc	35.2 ± 9.8 cd	49.9 ± 7.6 b	51.8± 6.9 b	49.1± 4.2 b	52 ± 5.8 b	33.4 ± 3.2 cd	33.9± 5.6 cd
NONANOL	fruity	> 0.05	40.9 ± 24.8 a	36.3 ± 23.1 a	54.9 ± 33.8 a	64.6 ± 33.7 a	64.5± 29.8 a	44.5± 28.5 a	54.9± 51.9 a	55.8 ± 44.4 a	52.7± 25.1 a	64.6 ± 34.1 a	42± 39.1 a	72.8± 49.9 a
BENZYL ALCOHOL	fruity	> 0.05	112.9 ± 57.9 a	122.6 ± 71.3 a	109.8 ± 36.2 a	125.7 ± 67.7 a	143.3± 80.2 a	81.6± 49.5 a	85.1± 49.8 a	107 ± 56.1 a	119.8± 52 a	129.8 ± 61.1 a	112.1± 34.9 a	112.7± 64.7 a
PHENYLETHYL ALCOHOL	rose	< 0.0001	44732.9 ± 3119.8 cd	60404.8 ± 3151.1 ab	46872.2 ± 3304.4 c	36660.6± 4446.7 de	34393 ± 2884.8 e	66045.9 ± 4192.8 a	46656.1 ± 3579.7 c	24811.1 ± 2948.7 f	28904.6 ± 3617.2 ef	31174 ± 2947.7 ef	56193.8 ± 3624.6 b	43073.2 ± 4685.7 cd
MCFA														
HEXANOIC ACID	sour	< 0.0001	12.6± 1.1 ab	11.4 ± 1.7 ab	12.8 ± 2.1 a	11.4± 2.4 ab	10.1 ± 1.8 ab	9.2± 0.5 b	9.8 ± 0.4 ab	11.4± 1.4 ab	9.7± 0.8 ab	11.1 ± 0.8 ab	9.5± 0.7 ab	12.1± 1 ab
OCTANOIC ACID	rancid	< 0.0001	5308± 796.3 a	4826.5± 880.8 a	5418.8 ± 946.4 a	4528.7 ± 1092.4 a	3984.9± 568.6 a	4061.8± 424.9 a	3747.4± 444.4 a	4229.6 ± 501.3 a	4059.5± 654.1 a	4481.8 ± 392.4 a	4633.6± 569.1 a	5465.3± 720 a
NONANOIC ACID		> 0.05	205.6± 137.6 a	73.7± 51 a	106.7 ± 57.9 a	172.5 ± 133.6 a	164.9± 55.2 a	130± 66 a	147.4± 49.5 a	154.8 ± 61.1 a	87± 58.8 a	148.6 ± 66.5 a	80.7± 57.5 a	152.8± 77.4 a
DECANOIC ACID	unpleasent	< 0.0001	687.9± 151.1 ab	441 ± 207.9 cd	573.8 ± 133.6 abcd	422.9± 153.7 d	512.1± 113.8 bcd	391.1± 167.9 d	405.6 ± 132.5 d	431.5± 136 d	482.7± 112.6bcd	476.6 ± 124.4 bcd	679.7 ± 142.1 abc	799.2± 111.8 a
9-DECENOIC ACID	waxy	< 0.0001	139± 30.4bcd	151.7± 79.4bcd	130.2 ± 22.6 cd	237.3± 45.4 ab	181.3± 30.9 bcd	88.5± 18.5 d	168.8± 36.8 bcd	175.9± 25.4 bcd	224.8 ± 59.2bc	328.4 ± 48.9 a	152.3± 25.3 bcd	117.5 ± 20.2 d
ALDEHYDES KETONES LACTONES														
BENZALDEHYDE	almond	> 0.05	34.2± 11.1 a	41.3± 8.8 a	34.6 ± 8.7 a	33.9 ± 10.6 a	30.9± 6.4 a	41.7± 8.9 a	33.5± 7.3 a	32.7 ± 7.3 a	34.8± 10 a	31.5 ± 7.5 a	41.4± 7.6 a	36.6± 8.2 a
TERPENES														
LINALOOL	rose	< 0.0001	21.1± 5.6abcd	14± 1.8 d	19.1 ± 3.8 abcd	22.7± 4.1 abc	19.5± 4.7abcd	19.9 ± 2.7abcd	16± 9.4 cd	26.1 ± 4.2 a	19.7 ± 2.4abcd	24.6 ± 4.4 ab	21.6 ± 3.2 abc	17.6± 4.1bcd
ESTERS														
ETHYL ACETATE	fruity	< 0.0001	421± 36.7 cd	607.1± 42.9 a	388.5± 19.1 d	450.3 ± 51.2 bcd	403.9± 42.5 cd	417.5 ± 56.6 cd	422.4± 29.2 cd	292.2± 30.6 e	428.3 ± 40 cd	475.1 ± 28.4 bcd	535.7± 82.8 ab	491.6± 42.8bc
ISOBUTYL ACETATE	fruity solvent	< 0.0001	3.4± 0.4ef	10.9 ± 1.3 ab	3.4 ± 0.2 ef	8.6± 1.7 bc	6.2± 1.1 cd	5.7± 1.1 de	7.4± 0.8 cd	2.4± 0.5 f	7.4 ± 1.3 cd	8.3 ± 1 c	5.4± 1.7 de	11.7± 1 a
ETHYL BUTYRATE	fruity	< 0.0001	32.5± 1.8 ab	27.8± 2.6bcd	30.3± 3.1 abc	37.1 ± 3.1 a	30.5± 5.2 abc	37.7± 3.9 a	28.1± 1.4bc	20.5± 4 d	33.7± 3.6 ab	33.9 ± 1.4 ab	23.6 ± 3 cd	37.5± 3.1 a
ISOAMYL ACETATE	banana	< 0.0001	3763.3 ± 224.8 d	9540.4± 815.8 a	3703.3± 200.2 d	5620.1 ± 713.4 c	4663.5± 736.2 cd	5093.4 ± 492.8 cd	5227.2± 460.7 c	1972.4± 417.8 e	5194.1± 547.3 c	4581.9 ± 541.9 cd	5621.9 ± 934.2 c	7115.2 ± 555.9 b
ETHYL HEXANOATE	fruity	< 0.0001	590.5± 57.8 abc	610.2± 60.3 abc	558.8 ± 55.4 abcd	571.6 ± 95.3 abcd	500.5± 111abcd	398.9± 69.7 d	602.6± 49.6 abc	464.1± 55.8 bcd	455.3 ± 48.2 cd	662.8 ± 33.3 a	464± 55.5 bcd	633.1± 41.3 ab
HEXYL ACETATE	banana	< 0.0001	117.4 ± 13.2 de	172.9± 18.5 abc	102.5± 14.4 e	140.1 ± 18.9 bcde	150.2± 33.4abcde	120.4 ± 23.4 cde	143.9± 4.8 bcde	37.6± 18.5 f	165.2 ± 16.7abcd	148.6 ± 3.9 abcde	173.2± 29.4 ab	199.1± 14.9 a
ETHYL HEPTANOATE		< 0.0001	0.2± 0.1 c	0.3± 0.1 c	0.2 ± 0.1 c	0.7± 0.3 b	0.3± 0 c	0.5 ± 0.2bc	0.8 ± 0.2 b	0.3± 0 c	0.4 ± 0.1bc	2.6 ± 0.3 a	0.3 ± 0.1 c	0.2± 0.2 c
HEPTYL ACETATE		< 0.0001	0.4 ± 0.1 d	0.8± 0.1 d	0.5± 0.2 d	3.3± 1.1 b	0.9 ± 0.2 d	0.7± 0.2 d	1.6± 0.2 cd	0.4± 0.1 d	2.4 ± 0.8bc	5 ± 0.7 a	0.8± 0.2 d	0.8 ± 0.4 d
METHYL HEXANOATE		< 0.0001	1.6 ± 0.1 b	2.4± 0.3 a	1.6± 0.1 b	0.6± 0.2 d	0.6 ± 0.1 d	1 ± 0.2 c	0.7± 0.1 cd	0.6± 0 cd	0.7 ± 0.2 cd	0.5 ± 0.2 d	0.5± 0.1 d	0.5 ± 0.2 d
ETHYL OCTANOATE	apricot . fruity	< 0.0001	171.1± 37.6 ab	221.3 ± 83.1 ab	142.7± 37.9 ab	162.1 ± 61.1 ab	280.8± 48.9 a	160.6± 74 ab	261.4 ± 59.4 ab	151.7± 16.5 ab	167.7± 91.3 ab	184.7 ± 95.8 ab	98.8± 38.3 b	111.1± 74.1 ab
ISOAMYL HEXANOATE	pineapple	< 0.0001	158.8± 33.4 ab	203.3 ± 73.8 ab	133.5± 33.7 ab	150.8 ± 54.3 ab	256.3± 43.4 a	149.5± 65.7 ab	239 ± 52.8 ab	141.5± 14.7 ab	155.7± 81.2 ab	170.8 ± 85.1 ab	94.5± 34 b	105.4± 65.9 ab
ETHYL NONANOATE		< 0.0001	2± 1 c	2.3 ± 0.8bc	1.4 ± 0.1 c	2.1± 0.5 c	5.1 ± 1.2 ab	2.4 ± 0.8bc	5.7± 3.6 a	1.8± 0.6 c	2.7 ± 2.2bc	3 ± 1.8 abc	1.1 ± 0.5 c	1.2± 0.6 c
ETHYL DECANOATE	sweet	< 0.0001	49.3± 5.7 ab	43.2± 5.6 b	40.7± 5.7 b	38.1± 10.7 b	71.5± 5.5 a	40.5± 10.3 b	54.7 ± 5.3 ab	42.8± 5 b	40.1± 14.2 b	42.2 ± 6.7 b	33.2± 8.6 b	31.2 ± 11.5 b
ISOAMYL OCTANOATE	sweet	< 0.0001	1.5± 0.3abcde	2± 0.5 abc	1.3 ± 0.3 bcde	1.2± 0.3 cde	2.2 ± 0.3 ab	1.7 ± 0.5abcd	2.3± 0.4 a	1.1± 0.1 de	1.1± 0.5 de	1.2 ± 0.3 bcde	0.9± 0.3 de	0.6± 0.3 e
ETHYL 9-DECENOATE	fruity	< 0.0001	32.9± 10.9 cde	43.2± 11.4bcde	24± 5.9 de	56.4 ± 16.1 abcd	71.4 ± 13.7 ab	29.4± 8.4 de	64.6± 8 abc	45.5± 3.5 bcde	52.3 ± 19.3abcd	82.6 ± 24.2 a	27.9± 11.8 de	13.4± 8 e
PHENYL ETHYL ACETATE	floral	< 0.0001	46.7± 4.3 e	148.6± 9.8 a	48 ± 6.4 e	45.8± 7.1 e	48.7 ± 5.2 de	75.2± 5.3 b	61.4± 5.8 cd	25.8± 2.7 f	42.4± 6.7 e	38.2 ± 2.2 ef	77.2± 5.4 b	64.4± 6.9bc
ETHYL DODECANOATE	waxy	< 0.0001	3.5± 0.8bc	6.5± 2.7 a	2.3 ± 0.3 bcd	2.3± 0.5 bcd	3.3± 0.8bc	4.1± 1.3 b	3± 0.3 bcd	2.3± 0.5 bcd	2.1 ± 0.5 cd	2.8 ± 0.7 bcd	1.7 ± 0.3 cd	1.2 ± 0.3 d
ETHYL MYRISTATE		< 0.0001	4.4± 1.2bc	14.8± 4.6 a	3.2 ± 0.9 c	2.2± 1.4c	3.3± 1.1 c	7.3± 3.7 b	2.9± 0.8 c	2.4± 0.6 c	2.2± 0.7 c	2.6 ± 0.7 c	2 ± 0.5 c	1.6± 0.5 c
ETHYL PALMITATE		< 0.0001	76.6± 31.7 ab	91.9± 38.1 a	49.4± 12.8 b	13.2± 6.9 c	16.7± 4.7 c	75.8± 28.1 ab	14.6± 2.4 c	8.9± 2.4 c	18.2± 7.2 c	12.9 ± 5.5 c	17.9 ± 6.6 c	10.1± 6 c
DIETHYL SUCCINATE		< 0.0001	0.2 ± 0.1 b	0.2± 0 b	0.2± 0.1 b	0.3± 0.1 b	0.3 ± 0.1 b	0.3± 0.1 b	0.4 ± 0.1 b	0.8 ± 0.1 a	0.3± 0.1 b	0.4 ± 0.2 b	0.2± 0 b	0.2 ± 0.1 b
